# Physiological and molecular insights into the resilience of biological nitrogen fixation to applied nitrogen in *Saccharum spontaneum*, wild progenitor of sugarcane

**DOI:** 10.3389/fpls.2022.1099701

**Published:** 2023-01-13

**Authors:** Ting Luo, Chang-Ning Li, Rui Yan, Kejun Huang, Yang-Rui Li, Xiao-Yan Liu, Prakash Lakshmanan

**Affiliations:** ^1^ Sugarcane Research Institute; Key Laboratory of Sugarcane Biotechnology and Genetic Improvement (Guangxi), Ministry of Agriculture and Rural Affairs, Guangxi Academy of Agricultural Sciences, Nanning, China; ^2^ Interdisciplinary Research Center for Agriculture Green Development in Yangtze River Basin, College of Resources and Environment, Southwest University, Chongqing, China; ^3^ Queensland Alliance for Agriculture and Food Innovation, University of Queensland, St Lucia, QLD, Australia

**Keywords:** hormone, nitrogen, fixation, *Saccharum spontaneum*, sugarcane

## Abstract

Excessive use of nitrogen (N) fertilizer for sugarcane cultivation is a significant cause of greenhouse gas emission. N use-efficiency (NUE) of sugarcane is relatively low, and considerable effort is now directed to exploit biological nitrogen fixation (BNF) in sugarcane. We hypothesize that genetic base-broadening of sugarcane using high-BNF *Saccharum spontaneum*, a wild progenitor of sugarcane, will help develop N-efficient varieties. We found remarkable genetic variation for BNF and growth in *S. spontaneum* accessions, and BNF in some accessions remained highly resilient to inorganic N application. Physiological and molecular analyses of two *S. spontaneum* accessions with high-BNF capacity and growth, namely G152 and G3, grown under N replete and low N conditions showed considerable similarity for total N, NH_4_-N, soluble sugar, indoleacetic acid, gibberellic acid, zeatin and abscisic acid content; yet, they were strikingly different at molecular level. Global gene expression analysis of G152 and G3 grown under contrasting N supply showed genotype effect explaining much of the gene expression variation observed. Differential gene expression analysis found an over-representation of carbohydrate and amino acid metabolism and transmembrane transport genes in G152 and an enrichment of lipid metabolism and single-organism processes genes in G3, suggesting that distinctly divergent metabolic strategies are driving N-related processes in these accessions. This was attested by the remarkable variation in carbon, N, amino acid and hormone metabolism-related gene expression in G152 and G3 under high- and low-N supply. We conclude that both accessions may be achieving similar BNF and growth phenotypes through overlapping but distinctly different biochemical and molecular mechanisms.

## 1 Introduction

Nitrogen (N) is one of the major essential elements for plant growth and development. Both carbon fixation and sugar production are directly affected by N deficiency in sugarcane (Robinson et al., 2008). In natural ecosystems, mineralization of organic pools provides the N used by the plants, and thus the growth of natural vegetation in general remains relatively low compared to those in managed agricultural ecosystems. Yet, to feed the growing world population, agriculture intensification through continuous high-input cropping became the norm for many crop production regions (Kopittke et al., 2019). This paradigm shift in crop cultivation driven by Green Revolution has made remarkable achievements in food production and food security. However, it also resulted in overuse of agro-chemicals causing severe unintended adverse impacts on environment and human health (Yang et al., 2021). Excessive use of agrochemicals, particularly inorganic N fertiliser, led to soil acidification, reduced soil fertility and crop productivity, ground water and air pollution, eutrophication and increased agriculture carbon footprint (Guo et al., 2010). Currently, about 120 Tg (million metric tonnes) of inorganic N is used annually worldwide (FAO, 2019) with 60-80% of it is lost to the environment due to a combination of excessive inorganic N fertiliser input and the low crop nitrogen use efficiency (NUE) (Robinson et al., 2014; Luo et al., 2022). The need for reduced N fertiliser input for sustainable crop production is now well recognised globally (Udvardi et al., 2021). To realise this outcome, it is also recognised that a multi-pronged crop production strategy involving transformational innovations in agronomy, increased use of organic sources of N including biologically fixed N, and improved crop genetics is needed (Rossetto et al., 2022; Luo et al., 2022).

Sugarcane is a fast-growing high-biomass crop cultivated in both tropical and sub-tropical countries. It provides most of the sugar and about 35% of ethanol produced globally (FAO, 2020; Yang et al., 2021). Sugarcane is mostly cultivated under rainfed condition, often in low fertile N-limited soils. It is highly responsive to N supply and, consequently, overuse of inorganic N fertiliser to boost cane yield is widespread (Robinson et al., 2011; Yang et al., 2022). For instance, N application rate for sugarcane ranges from 400 kg ha^−1^ in certain production regions in India to 1381 kg ha^−1^ in some areas of China, the two major sugarcane producing countries (Robinson et al., 2011; Yang et al., 2022). As with most other crops, NUE of sugarcane is relatively low, with crop recovering 20-40% of N applied even in well-managed production systems following best crop management practices (Luo et al., 2022). The substantial loss of applied N fertiliser to ground water, run off and atmosphere, and the attended environmental costs prompted considerable effort to improve sugarcane crop NUE in many cane producing countries. This include exploring the use of enhanced efficiency fertilisers, and increasing the soil organic N pool through crop residue retention, organic amendments such as sugar mill by-products and legume intercropping (Bell, 2014; Moreira et al., 2021). Also, considerable effort is now underway in researching and exploiting biological N fixation (BNF) to reduce inorganic N use for sustainable production of sugarcane and other crops (Solanki et al., 2020; Soumare et al., 2020).

Biological N fixation by diazotrophs, a diverse group of bacteria and archaea capable of fixing atmospheric N_2_ to NH_3_, is a significant source of N used by plants in different ecosystems, including agro-ecosystems (Solanki et al., 2020; Imran et al., 2021). Diazotrophs can be symbiotic endophytes as nodulating and non-nodulating bacteria or it can be associative diazotrophs inhabiting on rhizosphere and the bulk soil surrounding the root system, or on above-ground plant body (Imran et al., 2021). Growth improvement by rhizospheric and endophytic diazotrophs has been reported in sugarcane (Shastri et al., 2020; Singh et al., 2021a; Singh et al., 2021b). Under field condition, diazotrophs contribute up to 15% of crop N demand (Imran et al., 2021). In addition, they possess a number of plant growth promoting properties such as phytohormone production, solubilisation of minerals, control of pathogens, abiotic stress tolerance and siderophore formation in many crops including sugarcane (Singh et al., 2021b; Singh et al., 2021c). Reports from Brazil suggest that BNF accounts for a significant proportion, 60-80% in some cases, of N used by commercial sugarcane crops (Boddey et al., 1991). There are numerous reports of sugarcane BNF by associative diazotrophs from Brazil, China and India but attempts to detect sugarcane BNF in some other countries were unsuccessful. The reasons for the conflicting results are unclear, but strong host genotype specificity and soil environmental sensitivity of diazotrophs are now well-recognised (Imran et al., 2021). Considering the remarkable variation for host genotypic compatibility of diazotrophs (Malviya et al., 2022) and the inherent narrow genetic base of current commercial sugarcane varieties in general (Hemaprabha et al., 2022), we studied the abundance and diversity of rhizosphere diazotrophs in sugarcane ancestral species and found large rhizospheric microbial diversity in the analysed species (Malviya et al., 2022). Of the five wild *Saccharum* species analysed, *Saccharum spontaneum* is the most crossable, highly genetically diverse and ubiquitous in distribution. Being a versatile sugarcane progenitor with remarkable genetic diversity, we were interested in identifying accessions with high BNF capacity and desirable growth features useful for introgression breeding. As part of this research, we studied a population of 33 *S. spontaneum* accessions representing Chinese *Saccharum* spp germplasm collection covering very diverse tropical and sub-tropical ecological regions for their BNF property to identify accessions with high N fixing capacity. In most sugarcane production systems external N input is necessary for achieving economic yield. Because of this inevitability, we were interested in understanding the impact of externally applied inorganic N fertiliser on *S. spontaneum* BNF and how it affects carbon, N, amino acid and hormone metabolism at the molecular level. Hence, two *S. spontaneum* accessions with high BNF, well-developed stalk, high brix and flowering propensity were selected for further physiological and molecular studies to gain more insights into BNF and carbon, N, amino acid and hormone metabolism in this species, and the results are presented here.

## 2 Materials and methods

### 2.1 Plant materials and growing conditions

Wild accessions of sugarcane (*Saccharum* spp. interspecific hybrids) progenitor *Saccharum spontaneum* L. were used for this study. They were sourced from the Chinese *S. spontaneum* collection maintained in the germplasm garden of Sugarcane Research Institute, Guangxi Academy of Agricultural Sciences (SRI), Nanning, Guangxi, China. Nanning has a hot humid subtropical climate with annual temperatures ranging between 2°C and 35°C. It receives, on average, 1300 mm rainfall yearly with an annual mean humidity of 79%. For this study, thirty-three *S. spontaneum* accessions collected from very diverse tropical and sub-tropical agroclimatic conditions of Southern China, where sugarcane is grown commercially, were selected. All the selected accessions were free of pests and diseases, never fertilised, and grew well in the garden.

### 2.2 Screening of *S. spontaneum* population for BNF and growth attributes

Ten-month-old *S. spontaneum* plants were used for population screening experiment. Plants selected for the screening experiment were grown individually in large pots with soil collected from SRI germplasm garden in a naturally-lit glasshouse. They were not fertilised or sprayed with any chemicals prior to and during the screening experiment. The soil was irrigated as and when needed. The plants of all 33 accessions were divided equally into three blocks (six to eight plants of each accession in one replicate block), and in each block, they were arranged randomly. These accessions were screened for BNF activity using nitrogenase assay, and plant height (from soil surface to the dewlap of the youngest fully expanded leaf), stalk number and brix (using juice expressed from 10 cm of the basal part of stem) were also determined. Brix of expressed juice was measured by refractometry using ATR-P Refractometer (Schmidt and Haensch, Germany). For each accession there were six independent measurements from plants randomly selected from 3 replicate blocks.

#### 2.2.1 BNF assay: Nitrogenase activity of *S. spontaneum* accessions

Nitrogen fixation ability of *S. spontaneum* test clones was determined by assaying *in vivo* nitrogenase activity in nitrogen-free medium as described previously (Hardy et al., 1968). Lamina (1 g) of the youngest fully expanded leaf from each plant was sampled, cut into 0.5 cm long pieces and immediately transferred to 50 mL Erlenmeyer flask containing 10 mL of nitrogen-free assay medium. Then, under sterile condition, flask headspace air was removed and replaced with acetylene gas (10% v/v) and the flasks were incubated at 28 ^0^C for 48 h on a gyratory shaker set at 120 rpm. At the end of the incubation, 0.5 mL of headspace gas was removed from each flask and analyzed in a GC-17A gas chromatograph (Shimadzu, Japan) with DB-1,701 column (Agilent, Santa Clara, United States) using the flame ionization detector (FID) at 80 ^0^C and the injector at 110 ^0^C, with 35 mL min^−1^ flow rate of carrier gas. For each accession, nitrogenase activity of six individual plants selected randomly from 2 replicated blocks were determined. The amount of ethylene (C_2_H_4_) produced by each accession was calculated and presented as nmol C_2_H_4_ produced g^−1^ fresh weight h^−1^.

### 2.3 Impact of inorganic N fertiliser application on BNF in high-BNF *S. spontaneum* accessions

Six accessions with high BNF activity identified in the BNF screening experiment, namely G03, G152, G177, G720, G824 and G1926, were selected for studying the sensitivity of N fixation to high N condition. Single node cuttings from 12-month-old healthy plants were planted in unfertilized moist garden soil in plastic trays with perforated bottom and kept in a naturally-lit glasshouse for sprouting and plantlet development. One month after planting the cuttings, plantlets of uniform size were transplanted into 30 lit pots with unfertilized soil. All pots were watered regularly to avoid moisture stress. Two weeks after transplanting, potted plants were divided into two equal groups; one for high nitrogen (HN) and the other for low nitrogen (LN) treatments. All HN plants were supplied with Murashige and Skoog (MS) mineral nutrients (2 lit pot-1) (Murashige and Skoog, 1962), which contained 18.8 mM KNO_3_ and 20.6 mM NH_4_NO_3_, while those in LN received MS mineral nutrients without N (2 lit pot-1) once every three weeks. This N treatment was continued during the 5-month experimental period. The experiment followed a completely randomized block design with three replicated blocks. For both treatments, there were 5 plants of each accession in a single block (replicate). At the end of the experiment, middle portion of the youngest fully-expanded leaf was sampled and nitrogenase activity was determined as described above. For each accession, three biological replicates from each block were used for the enzyme assay.

### 2.4 Physiological and molecular responses of selected high-BNF *S. spontaneum* accessions G3 and G152 to inorganic N application

Two high-BNF *S. spontaneum* accessions with high brix, good stalk development, high stalk number, high propensity for flowering and a relatively low impact of applied inorganic N on their N fixation activity, namely G152 and G3, were selected for further physiological and molecular characterization. Plants were raised and the experiment was conducted as described in section 2.2. except for the following conditions. Experiment was continued for 7 months. The experiment followed a completely randomized block design with three replicated blocks for each treatment. Each block had 6-8 plants of each accession for HN and LN treatments.

At the end of the experiment, leaf tissue from the youngest fully expanded leaf and root samples were harvested for measuring nitrate reductase (NR) and glutamine synthetase (GS) activity, and total N, NH_4_-N, soluble protein and soluble sugars content. Leaf tissue was also used for BNF assay and transcriptome analysis. For NR and GS activity, lamina (1 gm) from the youngest fully-expanded leaf and root tips (10 mm from the tip) were collected and immediately snap-frozen in liquid nitrogen. The tissue samples were collected between 09.30 and 13.00 hours to minimise the effect of diurnal variation.

### 2.5 Shoot growth and content of total N, NH_4_-N and soluble sugars in G3 and G152 grown under externally supplied inorganic N

At the end of seven months of growth, shoot height and stalk number of experimental plants were measured. For chemical analyses, oven dried (80 ^0^C for 5 days) leaf and root tissues were finely powdered and used for total N, NH_4_-N and soluble sugars content measurement. Total N was determined by acid digestion of samples following Kjeldahl method. NH_4_-N and soluble sugars content were measured using Plant Ammonium Nitrogen Activity Assay Kit (# BC1520, Solarbio, China), and Plant Soluble Sugar Content Assay Kit (# BC0035, Solarbio, China), respectively, following manufacture’s instruction. For each treatment, eight independent plants (measurements) randomly selected from different replicated blocks were used for analysis.

#### 2.5.1 BNF activity of *S. spontaneum* accessions G3 and G152 grown under externally supplied inorganic N

Nitrogen fixation ability of *S. spontaneum* test clones supplied with and without N was determined by measuring *in vivo* nitrogenase activity of youngest fully expanded leaf tissue of seven-month-old plants in N-free medium as described in section 2.2.1.

#### 2.5.2 Nitrate reductase and glutamine synthetase activity of *S. spontaneum* accessions G3 and G152 grown under externally supplied inorganic N

Frozen leaf and root tissue samples were ground to a fine powder and used for nitrate reductase (NR, EC 1.7.1.1) and glutamine synthetase (GS, EC 6.3.1.2) activity assays. Five ml of the extraction buffer (50 mM Tris HCl, 1 mM MgSO4, 1 mM EDTA, 10 mM cysteine, 1% insoluble PVP) was mixed with 1 g of tissue and the homogenate was kept for 30 min on ice with occasional stirring. The homogenate was then centrifuged at 4°C at 12000 rpm for 10 min and the supernatant was used for enzyme activity measurement. The NR activity was measured by methods developed by Bories and Bories (1995), and expressed as the amount of NO_2_ produced g^−1^ fresh weight of tissue. The GS activity was measured according to Bressler and Ahmed (Bressler and Ahmed, 1984), and expressed as nmol γ-glutamyl-monohydroxamate (GHA) produced mg^−1^ protein min^−1^. Soluble protein content of sampled tissues was determined with bicinchoninic acid (BCA) method using bovine serum albumin as standard. For each treatment, eight independent plants randomly selected from different replicated blocks were used for analysis.

#### 2.5.3 Changes in endogenous level of plant hormones in G3 and G152 grown under externally supplied inorganic N

The frozen leaf samples of G3 and G152 plants grown with and without external N were finely powdered in liquid nitrogen. The powdered tissue (1 g) was extracted with cold methanol (10 ml) containing 1 mM butylated hydroxytoluene at 4°C for 16 h in dark as described previously (Gong et al., 2017). These samples were then centrifuged at 2000 rpm for 20 min at 4°C and the pH of supernatants collected was adjusted to 2.8, then extracted thrice with an equal volume of ethyl acetate, and the extract was evaporated to dryness in a vacuum centrifuge (RVC 2-25 CDplus, Christ, Germany). The dried samples were redissolved in 0.5 ml of methanol with 0.1M glacial acetic acid as the mobile phase for high performance liquid chromatography (HPLC) analysis. Hormones were quantified using RIGOL L-3000 HPLC system (RIGOL, Beijing, China) as described previously (Yang et al., 2014). Analysis was done using a Kromasil C18 column (250 mm*4.6 mm, 5 μm; EKA chemical Inc) with 100% methanol (A) and 0.1M acetic acid (B) as mobile phases and a flow rate set at 1 mL/min. Extracted samples (10 μL) were injected into column and gibberellic acid (GA_3_), abscisic acid (ABA), zeatin riboside (ZR) and indole-3-acetic acid (IAA) were detected at wavelengths 210 nm, 254 nm, and 275 nm, respectively. Plant hormone standards with known concentration were used for establishing calibration curves, which were used for quantifying hormones in the test samples.

### 2.6 Statistical analysis

All data presented here are analysed using analysis of variance (ANOVA) in Genstat statistical system, 19th Edition (VSNI, 2017).

### 2.7 Molecular analysis of high-BNF *S. spontaneum* accessions to understand carbon, N, amino acid and hormone metabolism-related gene expression

#### 2.7.1 Plant materials and RNA isolation

Youngest fully expanded leaves harvested from G152HN, G152LN, G3HN and G3LN plants (see section 2.4. for experimental details) were immediately snap-frozen in liquid nitrogen. Total RNA was isolated using TRIzol™ Reagent (Thermo Fisher Scientific, Wilmington, USA) and the RNA quality was monitored on 1% agarose gel. RNA purity was determined using the NanoPhotometer^®^ (IMPLEN, CA, USA). RNA samples were quantified spectrophotometrically using Qubit^®^ RNA Assay Kit in Qubit^®^ 2.0 Flurometer (Life Technologies, CA, USA) and RNA integrity was assessed using the RNA Nano 6000 Assay Kit of the Bioanalyzer 2100 system (Agilent Technologies, CA, USA). Two biological replicates were used for each treatment for molecular analyses.

#### 2.7.2 cDNA library construction and sequencing

A total of 1.5 μg RNA per sample was used for preparing the RNA for sequencing. Sequencing libraries were prepared using MGIEasy RNA library preparation kit (MGI, Shenzhen, China) and index codes were added to attribute sequences to each sample. PCR products were purified (MGIEasy DNA purification magnetic bead Kit) and the library quality was determined using the Agilent Bioanalyzer 2100 system. After purification, the double stranded PCR library was unzipped and then looped to form single stranded circular DNA. The rolling circle amplification (RCA) technology is used to form DNA nanoball (DNB), the DNB is loaded into the chip through the automatic sample loading system and fixed. The chip loaded with DNB was put into DNBSEQ-T7 for sequencing, and 150 bp double-ended sequencing reads was obtained. The transcriptome sequencing data are deposited into the National Center for Biotechnology Information (NCBI) SRA database under accession number PRJNA847754 and can be accessible with the following link https://www.ncbi.nlm.nih.gov/sra/PRJNA847754

#### 2.7.3 Data processing, transcriptome assembly and functional annotation

The raw reads in fastq format were initially processed through Perl scripts. After removing reads containing adapter and ploy-N, and low-quality reads, the clean reads obtained were used for Q20, Q30 and GC content. These high-quality clean reads were used for all the downstream analyses.

Clean reads were *de novo* assembled into transcriptome using Trinity v2.11.0 (Grabherr et al., 2011). Non-redundant unigenes were determined through sequence splicing and redundancy removal from all sample unigenes. The unigenes with lengths >200 bp were used for further analyses. All the assembled unigenes were searched and annotated using NCBI non-redundant protein sequences (Nr; https://www.ncbi.nlm.nih.gov/guide/), NCBI non-redundant nucleotide sequences (Nt; https://www.ncbi.nlm.nih.gov/guide/), Protein family (Pfam; http://www.pfam.org/), Gene Ontology (GO; http://geneontology.org/), Kyoto Encyclopedia of Genes and Genomes (KEGG) Orthology database (KO; https://www.genome.jp/kegg/ko.html), Swiss-Prot (https://web.expasy.org/docs/swiss-prot_guideline.html), and Clusters of Orthologous Groups of proteins (KOG/COG; ftp://ftp.ncbi.nih.gov/pub/COG/KOG), with an E-value cut-off of 1E-5.

#### 2.7.4 Analysis of differential gene expression in response to inorganic nitrogen application

The unigenes obtained were assembled into a Ref and the clean data for each sample were aligned back into the assembled Ref. Gene expression of all samples were calculated using RSEM v1.2.8. For the gene read counts of each library, DESeq2 v3.11 was used to estimate the transcripts per million values for each gene. Differential gene expression analysis of different comparative groups was performed using DESeq R package (1.18.0). DESeq provides statistical routines for determining differential expression in digital gene expression data using a model based on the negative binomial distribution. The resulting p -values were adjusted using the Benjamini and Hochberg’s approach for controlling the false discovery. Genes with an adjusted p-value < 0.05 and an absolute value of log2ratio (treatment/control) ≥ 1 were considered as differentially expressed genes (DEGs).

#### 2.7.5 GO and KEGG enrichment analysis

Gene Ontology (GO) enrichment analysis of differentially expressed genes was implemented by the GOseq R package, following gene length bias correction. GO terms with corrected p-value < 0.05 were considered significantly enriched by differential expressed genes. For KEGG pathway analysis we used KOBAS software to test the statistical enrichment of differential gene expression genes in KEGG pathways.

#### 2.7.6 Analysis of differential expression of genes involved in amino acids, nitrogen, carbon and hormone metabolism in response to N application

To expand our understanding how high N supply affects key metabolic pathways that regulate growth in *S. spontaneum* with BNF capacity we generated gene expression heatmaps and clustering of DEGs involved in carbon, nitrogen, amino acid and hormone metabolism of G3 and G152 accessions grown under external N supply. This analysis was performed with pheatmap R package using the FPKM value.

#### 2.7.7 Quantitative real time RT-PCR analysis

The RNA-Seq data was validated by quantitative real time RT-PCR analysis (qRT-PCR) using ten genes. The expression of eight selected genes were normalized using three reference genes, namely, glyceraldehyde-3-phosphate dehydrogenase (GAPDH); acyl-CoA dehydrogenase (ACAD);clathrin adaptor complex (CAC). The primers used for each gene are given in **Table S9**. The RNA for qRT-PCR was prepared as described in the section 2.7.1. cDNA was synthesized with HiScript II Q RT SuperMix (Vazyme, China) according to the manufacturer’s instructions. The qPCR reaction mixture (20 μL) consisted of 1 μL cDNA, 10 μL ChamQ Universal SYBR qPCR Master Mix (Vazyme, China), 0.4 μL forward primer, 0.4 μL reverse primer and 8.2 μL H20. The qPCR reaction followed a pre-denaturing step (95 °C for 3 min), amplification steps (95 °C for 20 s, 58 °C for 20 s, 72 °C for 25 s, and fluorescence acquisition) of 40 cycles, and a melting curves step (continue capturing fluorescence from 60°C to 95°C). The qPCR was performed on A qTOWER Real-Time Thermal Cyclers (Analytik Jena, Germany) was used for qPCR. The 2−ΔΔct method was used to calculate the relative gene expression.

## 3 Results

### 3.1 Substantial genetic variation for BNF, shoot growth, stalk number and brix exist in *S. spontaneum* accessions

**Figure 1:**
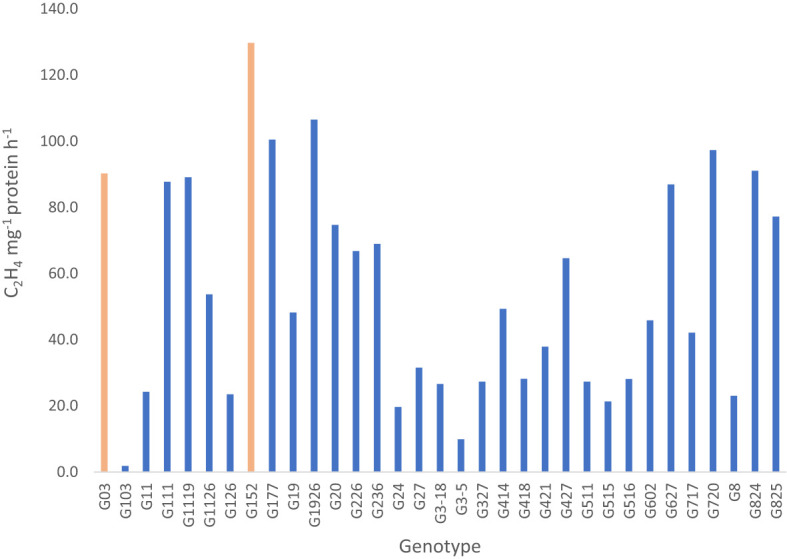
Nitrogenase activity measured by acetylene reduction assay of 33 *S. spontaneum* accessions showing large variation in their BNF activity. Values are mean of 6 independent measurements; l.s.d. 9.8, p < 0.001.

**Figure 2:**
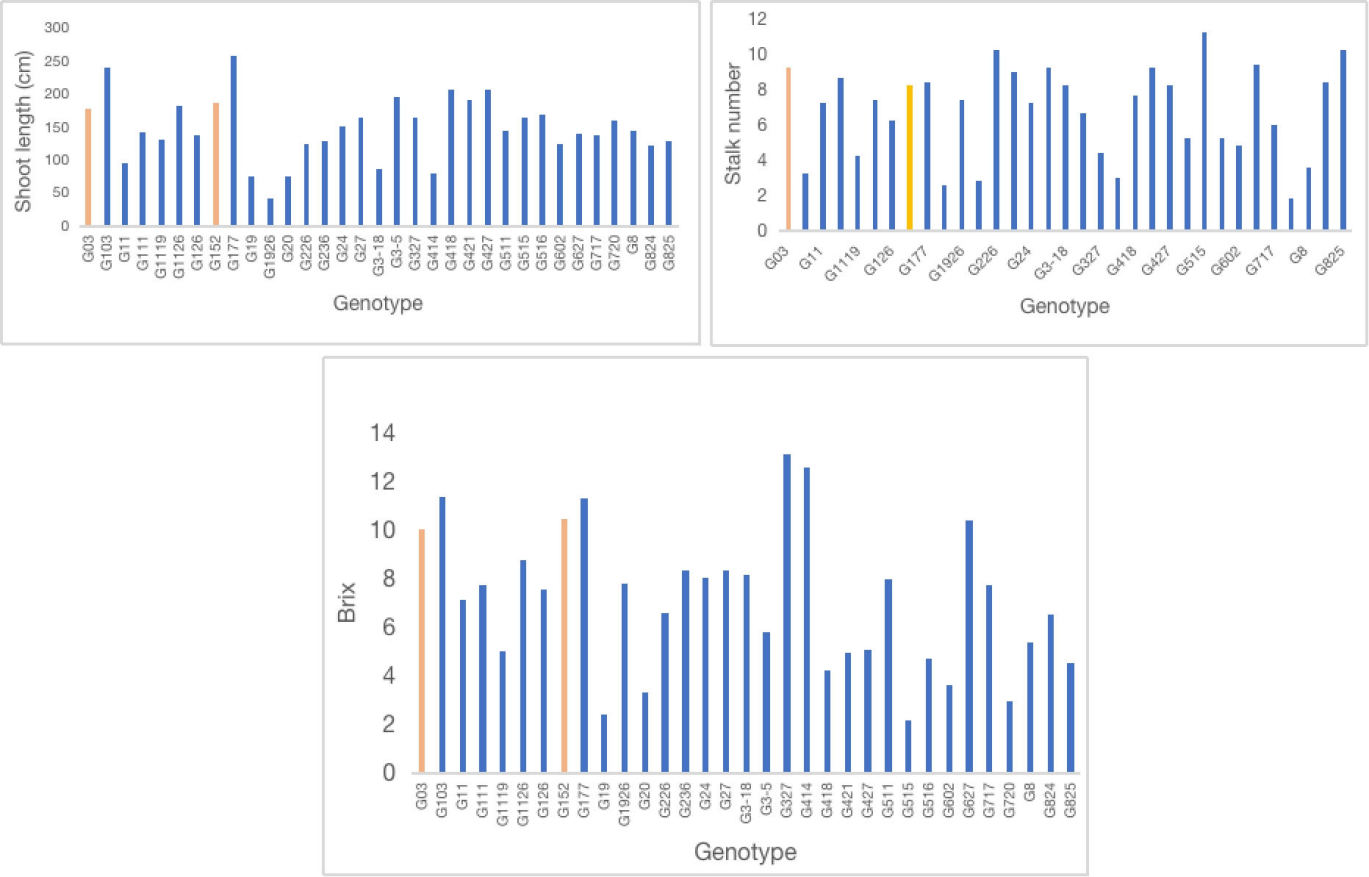
Variation in shoot length, stalk number per stool and brix (%) of *S. spontaneum* accessions Values are mean of 6 independent measurements; shoot length l.s.d. 15.4, p < 0.001; shoot number l.s.d. 1.2, p < 0.001; brix l.s.d. 1.09, p < 0.001.

**Figure 3:**
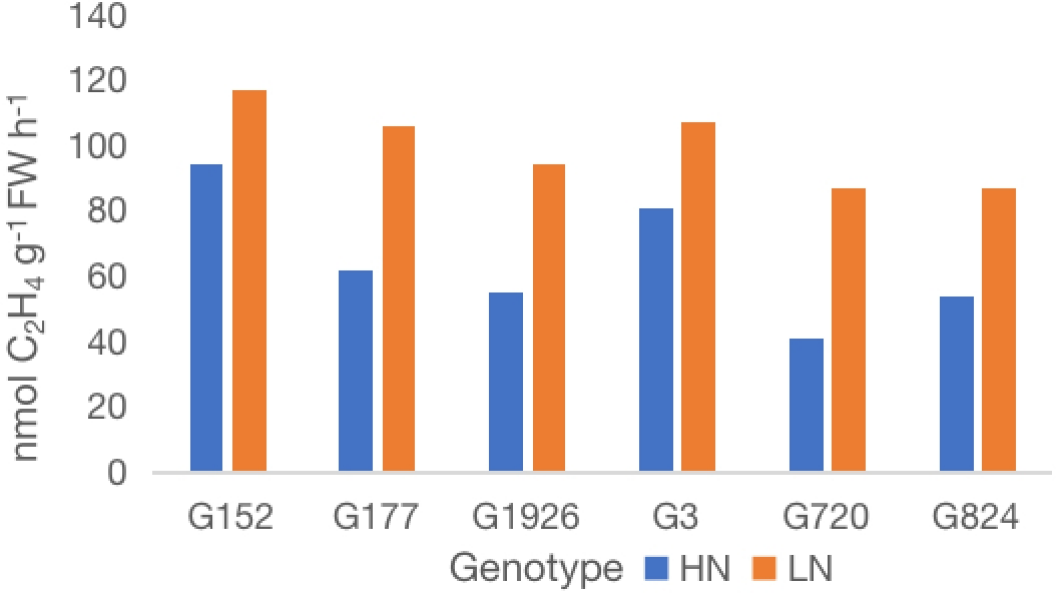
Effect of external application of inorganic N on nitrogenase activity of six high-BNF *S. spontaneum* accessions. Values are mean of 6 independent measurements; l.s.d. 12.4, p < 0.001. HN, high N; LN, low N.

Analysis of nitrogenase activity in *S. spontaneum* accessions showed remarkable variation for BNF capacity among the clones tested (**Figure 1**). For example, accession G152, the clone with the highest BNF activity, recorded 72-fold greater enzyme activity than the poorest performer, G103. Among the 33 clones tested, 15 had a relatively high BNF activity (>50 nmol C2H4 mg-1 protein h-1). A similar trend was also evident for plant height, stalk number and brix (**Figures 2**, **3**) although the range of variation for these traits was much smaller than that of BNF (**Figure 2**). On average, a six-fold variation was evident for plant height, stalk number and brix at the end of the experiment.

### 3.2 Sensitivity of BNF to inorganic nitrogen varies greatly among high-BNF *S. spontaneum* accessions

Analysis of nitrogenase activity in six high BNF accessions grown under externally supplied inorganic N showed remarkable genotypic variation for BNF activity (**Figure 3**). External application of inorganic N had a relatively low impact on the nitrogenase activity (20-25% reduction) of accessions G152 and G3, whereas the activity was reduced by 42-53% in other four accessions tested. The N-induced reduction in nitrogenase activity was significant for all accessions (p<0.01)

### 3.3 External nitrogen supply boosted shoot growth and content of total N, NH_4_-N and soluble sugars but reduced BNF in *S. spontaneum* accessions

**Figure 4:**
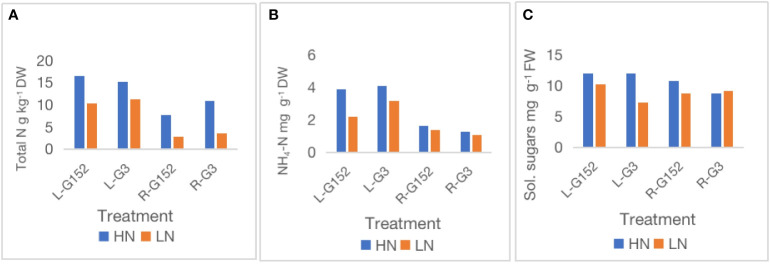
Effect of external application of inorganic N on leaf and root nitrogen **(A)**, NH4-N **(B)** and soluble sugars **(C)** content in *S. spontaneum* accessions G152 and G3. Values are mean of 6 independent measurements; L, leaf; R, root; HN, high N, low N, LN; total N l.s.d. 1.02, p < 0.001; NH4-N l.s.d. 0.52, p < 0.01; soluble sugars l.s.d. 0.64, p < 0.01.

External application of inorganic N significantly (p<0.01) increased shoot length and stalk number in both accessions (**Table 1**). Under external N supply, stalk length was increased by 19% in G3 and 22% in G152, whereas a more remarkable rise in stalk number, 46 – 50%, was observed in both accessions. Similar to shoot growth, total N and NH4-N content of leaf and root tissues of G152 and G3 accessions also showed a substantial increase when plants received external N supply (**Figures 4A, B**). Total N content variation was more pronounced in roots than in leaf tissue for both accessions, but that was not the case for NH4-N; there was very little variation for root NH4-N in G152 and G3plants. Compared with total N content, N-induced increase in soluble sugar was markedly lower in leaf and root tissues except for G3 leaf tissue (**Figure 4C**).

**Table 1:** Effect of external application of inorganic N on stalk length and stalk number of *S. spontaneum* accessions G3 and G152.

Treatments	Stalk length (cm)	Stalk number stool^-1^
G3HN	214	12
G3LN	177	9.2
G152HN	231	14
G152LN	186	8.2
l.s.d.	19.6	2.4
	P<0.01	P<0.01

Values are mean of 6 independent measurements. HN, high N; LN, low N.

**Figure 5:**
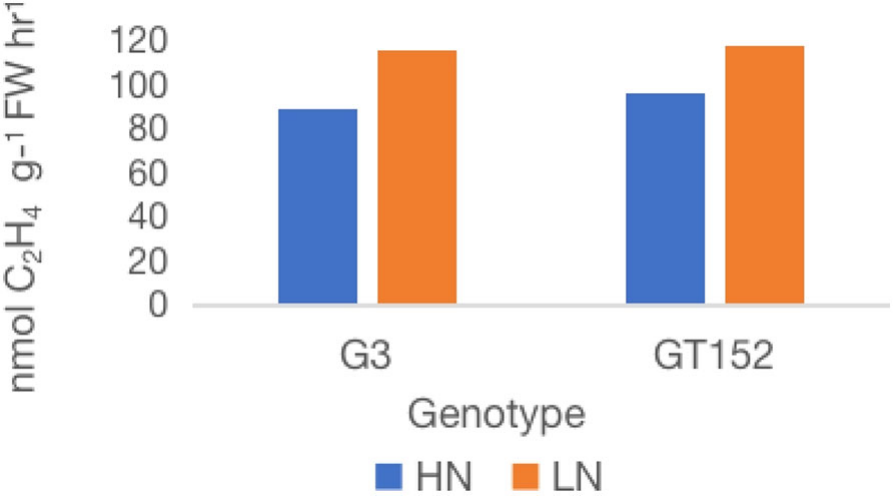
Effect of external application of inorganic N on nitrogenase activity of *S. spontaneum* accessions G152 and G3. Values are mean of 6 independent measurements; l.s.d. 7.3, p < 0.01. HN, high N; LN, low N.

From the six high-BNF accessions tested for their BNF-response to externally applied inorganic N (**Figure 3**), two accessions with BNF least affected by external N and desirable agronomic features as well as flowering propensity, G152 and G3, were selected for more detailed physiological and molecular analyses. Consistent with the results of the previous experiment (**Figure 3**), external application of N significantly reduced (19-23%; P<0.001) the nitrogenase activity in both accessions (**Figure 5**).

**Figure 6:**
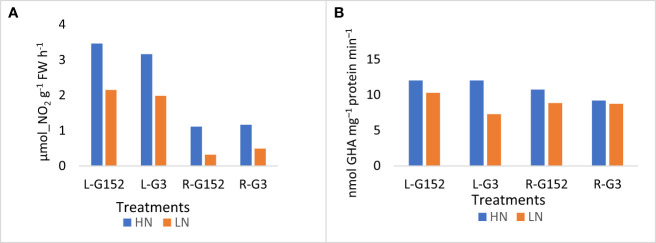
Effect of external application of inorganic N on leaf and root nitrogenase **(A)** and glutamine synthetase **(B)** activity in *S. spontaneum* accessions G152 and G3. Values are mean of 6 independent measurements; NR Ls.d. 0.58, p < 0.01; GS l.s.d. 1.29, p < 0.001; L, leaf; R, root; HN, high N; LN, low N.

Activity of NR in both leaf and root tissues of G152 and G3 accessions was substantially increased with the application of inorganic N (**Figure 6A**; p<0.001). The percentage increase in roots was much greater (2.4 to 3.5-fold) than that of leaf, and in both accessions rate of enzyme induction in leaf tissue was somewhat similar (~1.5 fold) (**Figure 6A**). As with NR, GS activity also showed an increasing trend in leaf and root tissues though the N-induced enzyme induction was not as pronounced as in NR, except for the G3 leaf tissue (**Figure 6B**).

### 3.4 Nitrogen supply increased auxin, gibberellin and cytokinin content and nitrate reductase activity remarkably, but not so for abscisic acid and glutamine synthetase

Inorganic N application had a remarkable effect on endogenous hormones in both G152 and G3 accessions (**Table 2**). The endogenous level of growth promoting hormones IAA, GA3 and ZR in both accessions increased by 60-80%, except for a doubling of ZR in G152. No consistent and significant change in ABA content was noticed with N application.

**Table 2:** Effect of external application of inorganic N on endogenous levels of hormones in the leaf tissue of *S. spontaneum* accessions G152 and G3.

Treatment	IAA (ng/g FW)	GA3(μg/g FW)	ABA (μg/g FW)	ZR (μg/g FW)
GSM152 HN	73.1	1.83	0.19	1.23
GSM152 LN	41.1	1.1	0.24	0.58
GSM3 HN	126.5	1.34	0.26	1.09
GSM3 LN	73.3	0.79	0.22	0.68
l.s.d. (5% level)	7.1	0.2	0.01	0.11
p	<0.01	<0.01	<0.004	0.001

Values are mean of 6 independent measurements. IAA, indole-3-acetic acid; GA3, gibberellic acid; ABA, abscisic acid; ZR, zeatin riboside.

### 3.5 RNA sequencing, *de novo* assembly of reads and functional annotation of unigenes

In the RNA-Seq experiment, the number of clean reads obtained from each library after trimming and filtering ranged between 44725188 and 481766948, with average 6.83 (range 6.09-7.07) gigabases, 93.6% Q30 (range 91.82-94.4%) and 53.5% GC (range 50.48-55.07%) content. Out of 266228 transcripts obtained, 110947 unigenes with a mean length of 1087 bp (range 201- 17867 bp) were identified (**Table S1**).

The function of all unigenes obtained were annotated using seven databases. (**Table S2–S4**). In this analysis, transcriptome assembled from RNA-Seq data was used as reference. Out of a total of 110947 unigenes identified, 64.9% (72056), the largest proportion of unigenes, were annotated from NT, followed by 67404 (60.8%) in NR. The lowest number of genes annotated, 8435 (7.6%), was in KOG. It is important to note that 76.6% (84986) of all the unigenes identified were annotated at least in one of the seven databases used in this study.

### 3.6 Differential gene expression in high-BNF *S. spontaneum* accessions grown under external N supply: Genotype effect far outweighed treatment effect

In the gene expression study, the impact of external application of N on the expression of genes associated with carbon, N, amino acids and hormones in leaf tissues of two high-BNF *S. spontaneum* accessions, G152 and G3, was analysed. Four different pair-wise comparisons, i.e., same accession with contrasting N treatments, high N and low N, comparison (G152 HN *vs* G152 LN; G3HN *vs* G3 LN) and different accessions with the same N treatment comparison (G152 HN *vs* G3 HN; G152 LN *vs* G3 LN), were performed to identify DEGs (**Figure S1**). In the initial global analysis of differential gene expression using the threshold of an adjusted p value of <0.05 and log2FoldChange >1 based on DESeq2 method, 864 genes were found to be differentially expressed between G152HN and G152LN with 360 genes downregulated and 504 upregulated (**Figure S1**). For the same comparison (HN *vs* LN) in G3, the number of DEGs was less than half of what was found in G152 (HN *vs* LN), and more genes were downregulated (155) than upregulated (87). In contrast to treatment comparison (HN *vs* LN for the same genotype), the genotype comparison (G152HN *vs* G3HN, G152LN *vs* G3LN) showed a substantially large number of DEGs, >2000 in each comparison, irrespective of the treatment (**Figure S2**). However, the pattern of gene expression in genotype comparison under high N treatment (G152HN *vs* G3HN) was just opposite of low N treatment (G152 LN *vs* G3 LN) with more downregulated DEGs under HN while the opposite was true for LN.

In order to identify common and unique genes that expressed differentially in response to N application in G152 and G3 accessions, Venn diagrams of DEGs were prepared (**Figure S2**). There were 28 common DEGs among G152 and G3 for treatment (HN *vs* LN) comparisons (**Figure S2A**), In this comparison (HN *vs* LN), G152 accession showed four-fold greater DEGs than that of G3 (**Figure S2A**). However, when G152 and G3 accessions were grown under similar soil N condition (**Figure S2B**), 1101 common DEGs were found. And, a similar number of DEGs was also found for genotype comparison under HN and LN conditions (**Figure S2B**). Interestingly, there were no common DEGs across all pair-wise comparisons (**Figure S2C**).

### 3.7 Carbohydrate, amino acid and energy metabolism DEGs over-represented in G152 in response to external N, while lipid, secondary metabolites and carbohydrate metabolism dominated in G3

**Figure 7:**
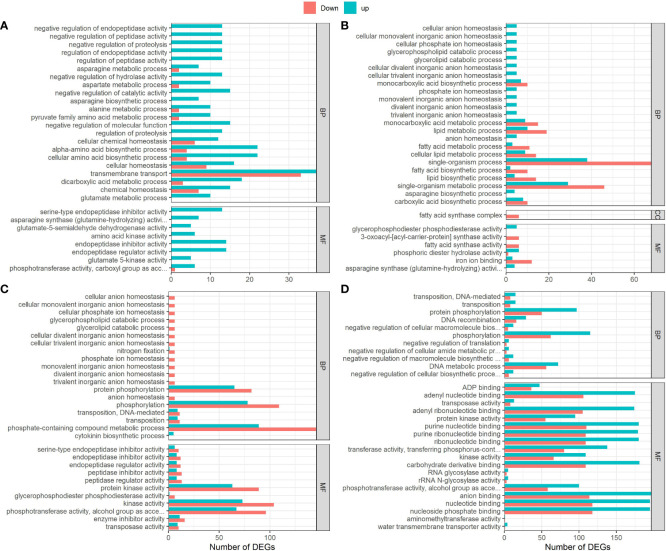
Gene ontology (GO) annotation of DEGs in *S. spontaneum* accessions G152 and G3 grown in high and low N treatments. The DEGs used here were identified by pairwise comparisons between high N and low N treatments and between genotypes (p-value < 0.05). **(A)**, G152HN vs G152LN; **(B)**, G3HN vs G3LN; **(C)**, G152 LN vs G3 LN; **(D)**, G152 HN vs G3 HN.

To determine the potential functions of DEGs identified in this study, we performed gene ontology (GO) enrichment analysis and they were grouped into two main GO functional categories- biological processes and molecular functions. There was large variation for functional classes between all four pair-wise comparisons (**Figure 7**). For instance, DEGs identified in G152 HN *vs* LN comparison were mostly mapped to regulation of proteolysis, peptidases, catalytic activity, amino acid, protein and carboxylic acid metabolism and membrane transport GO terms in biological process and molecular functions categories combined (**Figure 7A**). Most of the DEGs in this comparison were upregulated with transmembrane transport and amino acid biosynthesis being the most enriched ones. In contrast, DEGs found in G3HN *vs* LN comparison were enriched for single-organism process, fatty acid, lipid and carboxylic acid metabolism, and anion homeostasis for biological processes, and fatty acid and iron binding for molecular functions (**Figure 7B**). Another interesting observation noted in this analysis was that when both accessions grown under the same N supply conditions (HN or LN) were compared, a substantially greater number of DEGs was highly enriched in samples from HN than those from LN (**Figures 7C, D**). Also, in the genotype comparison under HN condition, DEGs were mostly up-regulated whereas an opposite trend was true for LN plants (**Figures 7C, D**). Under LN condition, highly enriched DEGs were attributed to phosphorylation and metabolism of phosphorous-containing compounds GO terms in biological processes category, while protein kinase, transferases form the most enriched GO terms in molecular functions category. In the comparison of genotypes grown under HN, highly enriched DEGs identified were mapped to phosphorylation and DNA metabolic processes for biological processes, and nucleotide binding, kinases, carbohydrate derivative-binding, phosphotransferases, and anion binding, for molecular processes (**Figures 7C, D**). Also, it is interesting to note that under LN growth condition, though only minimally enriched, cytokinin biosynthesis was up-regulated (**Figure 7C**).

**Figure 8:**
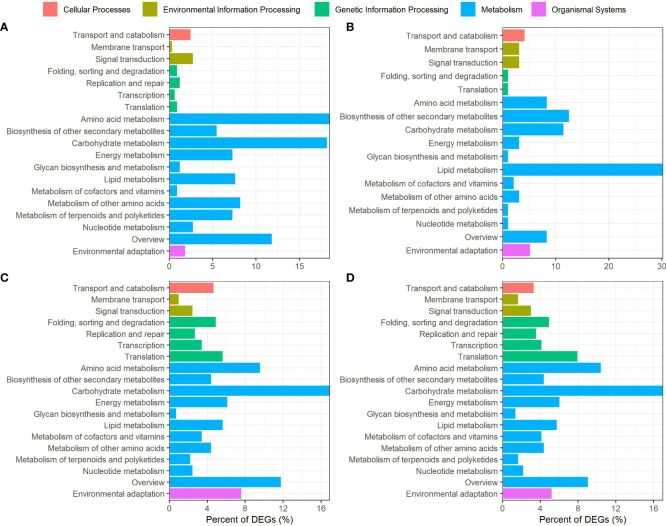
Kyoto Encyclopedia of Genes and Genomes (KEGG) pathways analysis of *S. spontaneum* accessions G152 and G3 grown under high N and low N conditions. The DEGs used here were identified by pairwise comparisons between high N and low N treatments and between genotypes (p-value < 0.05). **(A)**, G152HN vs G152LN; **(B)**, G3HN vs G3LN; **(B)**, G152 LN vs G3 LN; **(D)**, G152 HN vs G3 HN.

In order to gain more insights into the potential metabolic roles of genes differentially expressed in G152 and G3 accessions, they were mapped to various metabolic pathways in KEGG database (**Figure 8**). The genes differentially expressed in G152 in response to external N supply were mostly mapped to metabolism category with amino acid metabolism and carbohydrate metabolism being the most enriched pathways followed by lipid, energy, other amino acids, terpenoids and polyketides metabolism and biosynthesis of secondary metabolites (**Figure 8A**). Transport and catabolism, and signal transduction in cellular processes and environmental information processing categories, respectively, were the other significant pathways identified in G152 HN *vs* G152 LN comparison (**Figure 8A**). As with G152, DEGs identified in G3 HN *vs* LN comparison were mostly over-represented in metabolism category (**Figure 8B**). However, in G3, DEGs were most enriched for lipid metabolism pathway followed by biosynthesis of secondary metabolites and carbohydrate and amino acid metabolism pathways. Transport and catabolism, membrane transport and signal transduction pathways were also significantly enriched.

Unlike the large variation in representation of DEGs in various KEGG metabolic pathways seen between G152 and G3 grown under contrasting N supply (HN *vs* LN comparison) (**Figures 8A, B**), there were remarkable similarity for enrichment of DEGs in G152 and G3 grown under same N level (HN or LN) (**Figures 8C, D**). Carbohydrate metabolism was the most over-represented category in this genotype comparison, followed by amino acid, energy, lipid and nucleotide metabolism, and biosynthesis of secondary metabolites. Significantly, DEGs mapped to component pathways of genetic information processing category and that of transport and catabolism, and environmental adaptation, showed greater representation in genotype comparison (**Figures 8C, D**) than in treatment comparison (**Figures 8A, B**).

### 3.8 Distinct genotype- and N-dependent differential expression of genes involved in amino acids, carbon, nitrogen and hormones metabolism

**Figure 9:**
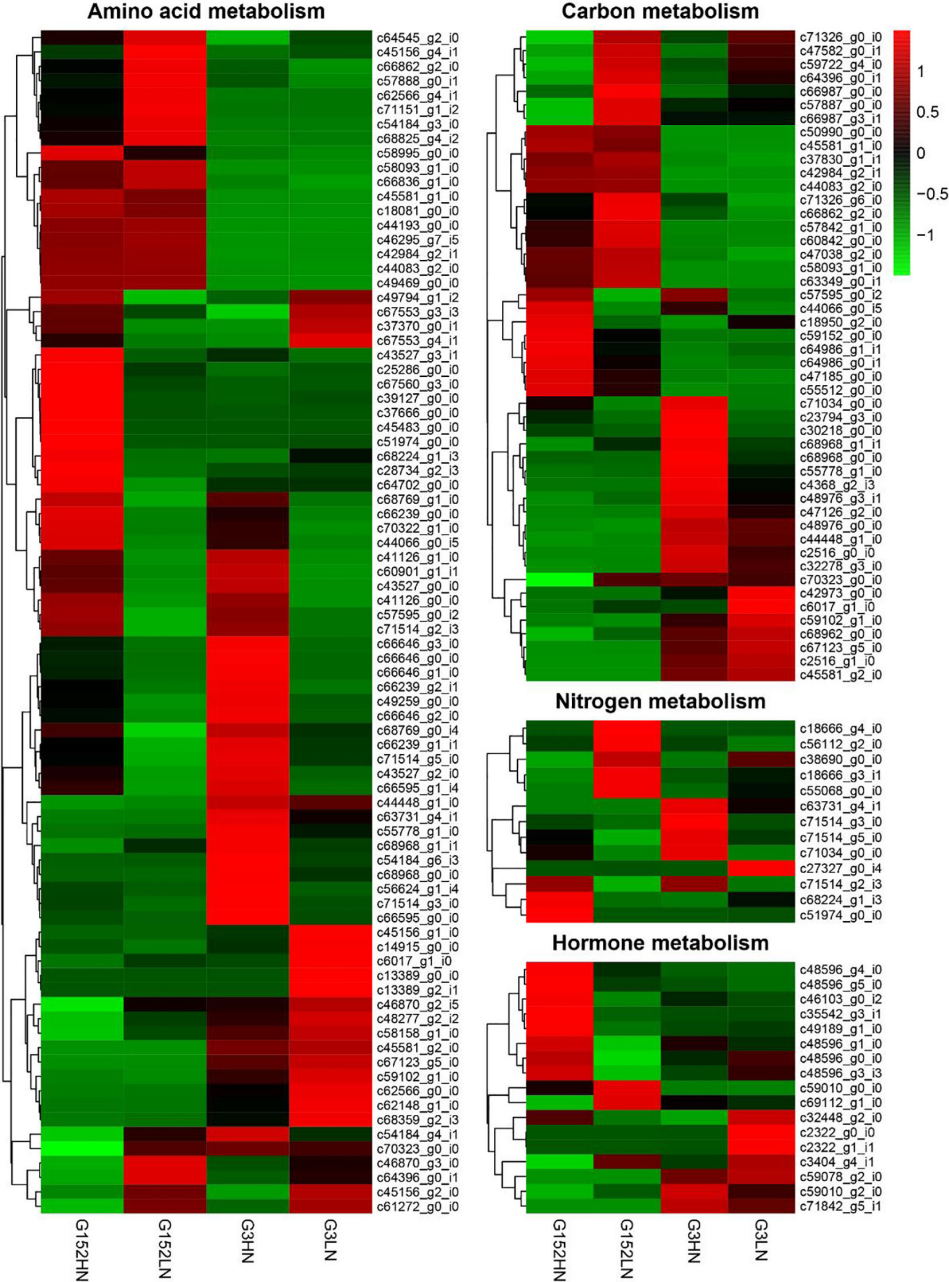
Expression of genes of involved in amino acids, carbon, nitrogen and hormones in response to external N application in *S. spontaneum* accessions G152 and G3.

Expression of DEGs involved in amino acid, carbon, N and hormones were further studied to gain more insights into metabolic responses of G152 and G3 accessions to inorganic N fertiliser application (**Figure 9**). A large number of amino acid metabolism-related genes were differentially expressed in response to external N supply in both accessions (**Figure 9**; **Table S5**). Also, there was substantial genotypic variation for this gene expression response. For instance, many amino acid metabolism-related genes that are up-regulated in G152 under high N condition were either down-regulated or remained unchanged under low N, and vice versa (**Figure 9**). This include glutamine synthetase, asparagine synthase, alanine transaminase, glutamate synthase, tryptophane synthase, etc. A similar result was also found for G3 grown under HN and LN conditions (**Figure 9**; **Table S5**). Several genes up-regulated in G3 under HN, such as alanine transaminase, glutamate dehydrogenase, glutamate synthase, asparagine synthase and serine acetyltransferase were either down-regulated or remained unchanged under LN condition. A similar notable result was the substantial variation in amino acid gene expression observed between G152 and G3 grown under same N condition (HN or LN). For instance, caffeoyl-CoA O-methyltransferase gene activity did not change in G152HN but it was down-regulated in G3HN. Proline dehydrogenase gene was up-regulated in G152 LN but its activity hardly changed in G3 LN (**Figure 9**). There was little change in the expression of serine O-acetyltransferase in G152HN but was over-expressed in G3HN, and it was down-regulated in G152LN with little change in G3LN. Collectively, these results show large N-induced and genotype-dependent variation for amino acid metabolism in the *S. spontaneum* accessions tested.

As with amino acid metabolism, expression of DEGs involved in carbon metabolism changed significantly in G152 and G3 accessions in response to inorganic N fertiliser application (**Figure 9**, **Table S6**). For example, in both G152 and G3, genes encoding fructose-bisphosphate aldolase, phosphoglycerate kinase and pyruvate dehydrogenase were down-regulated under HN condition whereas, under LN condition, they were over-expressed in G152 but remained unchanged in G3 (**Figure 9**, **Table S6**). In contrast, phosphenolpyruvate carboxylase, 6-phosphofructokinase 1 and ribulose phosphate 3-epimerase were over-expressed in G152HN but its expression did not change under LN condition. However, all these three enzymes were down-regulated in G3 irrespective of its soil N condition. Similar to amino acid metabolism, remarkably opposite expression patterns of DEGs were observed between G152 and G3 grown under HN or LN condition. This further attests the fact that genotypic variation is remarkably greater than treatment effect for carbon metabolism in the test accessions.

Consistent with the activity of amino acid and carbohydrate metabolism genes, expression of DEGs involved in N metabolism also showed remarkable variation under contrasting N supply in G152 and G3 accessions (**Figure 9**, **Table S7**). As an example, expression of nitrate and nitrite transporters and nitrate reductase genes was mostly inhibited in G152 and G3 grown under high N condition whereas their activity under low N was strongly up-regulated in G152 but remained mostly unaffected in G3. Contrarily, glutamate synthase expression was not affected in G152HN but was down-regulated in G3HN.The gene activity was either down-regulated or unaffected under LN condition in both accessions. In general, N metabolism gene expression showed remarkable variation in different genetic background and contrasting soil N conditions.

Genes involved in plant hormones auxin, gibberellin, cytokinin, abscisic acid and brassinosteroid metabolism were substantially altered by externally applied inorganic N in high-BNF *S. spontaneum* accessions G152 and G3 (**Figure 9**, **Table S8**). Under high N supply condition, (+)-abscisic acid 8’-hydroxylase gene expression was up-regulated conspicuously in G152 but it remained unchanged or down-regulated under low N supply in G152, and in G3 in both soil N conditions. A similar observation was also evident for GA2-oxidase except for G152LN plants where the gene activity was down-regulated. With regard to auxin metabolism, auxin-responsive GH3 genes involved in auxin homeostasis were slightly down-regulated in G152HN, G152 LN and G3HN plants but were up-regulated in G3LN (**Figure 9**, **Table S8**). A somewhat similar trend was also observed for transcripts of cytokinin dehydrogenase, a gene regulating cytokinin homeostasis. In contrast, a cytochrome P450 gene involved in brassinosteroid biosynthesis was strongly up-regulated in G152HN plants but not in plants from the other three treatments. In brief, as with external N treatment, genotype also had a pronounced effect on hormone metabolism in the test plants.

### 3.9 Expression of DEGs tallied well with qRT-PCR results

**Figure 10:**
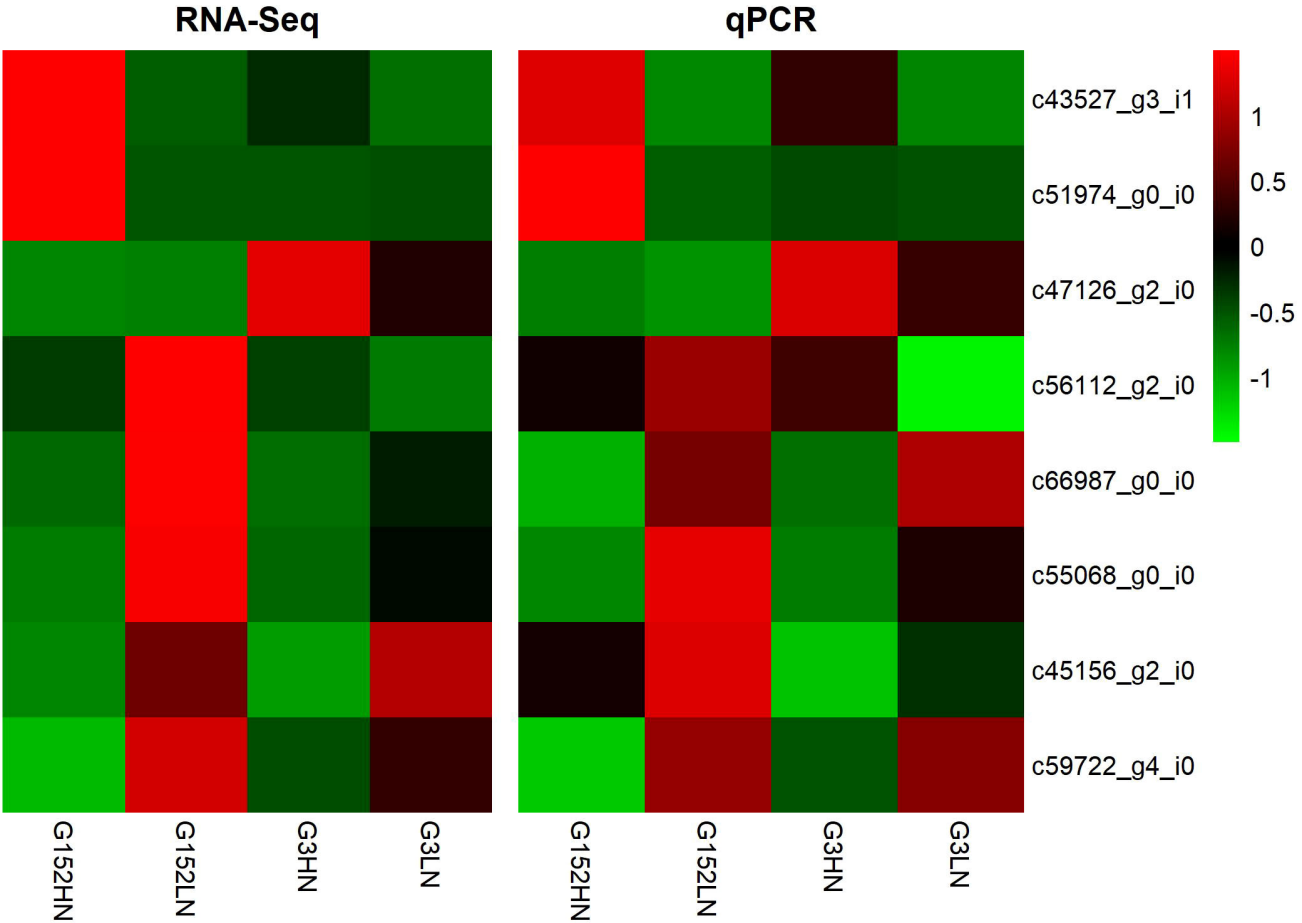
Validation of RNA-Seq data by analysing the expression of nine genes involved in amino acid, carbon and nitrogen metabolism by qRT-PCR.

The reliability of RNA-Seq data was confirmed by qRT-PCR analysis of 8 randomly selected unigenes involved in amino acid (c43527_g3_i1; c45156_g2_i0), carbon (c47126_g2_i0; c59722_g4_i0; c66987_g0_i0) and nitrogen metabolism (c51974_g0_i0; c55068_g0_i0; c56112_g2_i0) (**Figure 10**). The expression data from RNA-Seq was consistent with qRT-PCR except for a few minor quantitative variations.

## 4 Discussion

One of the important and novel results of this study is the finding that large genetic variation for BNF and brix exists in *S. spontaneum* accessions (**Figures 1** - **3**). And, some of the accessions have remarkably high capacity for BNF and favourable stalk traits including brix content (~10%). This result has considerable practical significance in that the native N fixing property of *S. spontaneum* could be introgressed into commercial sugarcane breeding population, similar to disease resistance, abiotic stress tolerance, vigour, ratooning, stalk population traits previously introduced (Lakshmanan et al., 2022). There are very few reports on BNF in *S. spontaneum* (Dong et al., 2018; Malviya et al., 2022), and extensive screening of geographically diverse accessions for BNF has not been reported. The data presented here forms the first evidence that high-BNF *S. spontaneum* with desirable agronomic attributes could be a potentially valuable genetic source for sugarcane BNF improvement through breeding. This is particularly relevant at present as sugarcane is touted as a sustainable food and energy crop (Yang et al., 2021; Camargo et al., 2022). However, its sustainability and carbon neutrality remain unclear (Yang et al., 2021; Camargo et al., 2022; Luo et al., 2022). As an example, China is a major sugarcane producing country and the excessive use of N fertiliser in sugarcane crop for a long period of time led to extensive soil degradation, soil and water pollution and crop productivity plateau (Luo et al., 2022). Thus, minimising fertiliser input, particularly N fertiliser use, for sugarcane production is critical for controlling soil degradation, improving crop NUE and regaining soil health and crop productivity.

While the large genetic variation for BNF in Chinese *S. spontaneum* is promising, little is known about its sensitivity to external N. This knowledge is very important for *S. spontaneum* introgression to improve NUE because significant quantities of inorganic N supply is needed to sustain economic yield in almost all sugarcane growing countries and any change in N input reduction is likely to be gradual. Also, BNF alone cannot meet crop N demand and sustain economic yield as observed in Brazilian sugarcane production. Hence, we studied the sensitivity of N fixation to externally applied inorganic N in high-BNF *S. spontaneum* accessions. The results showed remarkable resilience of BNF to relatively high externally supplied inorganic N in two (G152 and G3) out of six accessions grown in soil (**Figure 4**), demonstrating the potential of *S. spontaneum* for reducing external N requirement for sugarcane production through variety improvement (Liu et al., 2020; Udvardi et al., 2021).

Externally applied N is known to inhibit BNF even in high-N fixers like legumes (Liu et al., 2020; Imran et al., 2021; Udvardi et al., 2021). This is not surprising in that ready availability of fixed N inhibits N fixation (Udvardi et al., 2021). Hence, more experiments were conducted to further understand the physiological and molecular aspects of external N effect on BNF in *S. spontaneum* accessions G152 and G3. It appears that external N supply affects N and carbon metabolism differently in different accessions and even in different tissues. For example, external N supply inhibited BNF in both accessions but it greatly increased total N content of leaf and root tissues (**Figure 5A**). However, a different pattern was evident for NH4-N and soluble sugars with external N increasing their contents in leaf tissues of G152 and G3 but not so in the roots. This indicates that the effect of external N supply on carbon and N metabolism in *S. spontaneum* is organ-specific with a remarkable effect on leaves but has a minimal impact on roots (**Figure 5**). Such organ- and tissue-specific variation on carbon and N metabolism has been reported previously (Lawlor, 2002). Here, it is also interesting to note that different N metabolic enzymes responded differently to external N supply. For instance, NR activity was upregulated markedly in leaf and root tissues in response to external N in both varieties but GS activity was not much affected (**Figure 6**). Differential organ-specific expression of NR and GS enzymes in response to external N supply has been reported in other crops (Prinsi and Espen, 2015; Iqbal et al., 2020). Further, it is worth noting that the upregulation of N metabolism enzymes in leaf tissues of G152 and G3 was opposite of BNF activity. Collectively, these results suggest that external N supply may be regulating N and carbon metabolism differently in different organs and genotypes, and key N metabolism enzymes within each organ, in *S. spontaneum*.

Hormones regulate all aspects of plant growth and development. And, it is well established that nutrient availability, particularly macro-nutrients, determine plant growth and development (Krouk et al., 2011). It is also now well-established that plant growth, hormones and plant nutrition are finely coordinated through a network of hormonal and nutritional signal (Krouk et al., 2011). Since carbon and N are very central to growth and development of every organism, it is not surprising that its uptake, use and storage in plants are under hormonal regulation (Krouk et al., 2011; Yu et al., 2016). From the results of our study, it is clear that external N supply markedly increased plant growth promoting hormones such as auxin (IAA), gibberellin (GA3), and cytokinin (ZR) with little effect on abscisic acid in the leaf tissues of both varieties (**Table 2**). These results are corresponding well with the increased shoot elongation growth and shoot number observed in plants grown with external N supply (**Table 1**). Modulation of nutrient uptake, its use and remobilization, and plant grown by externally applied hormones, especially plant growth promoters, has been observed in many plant species (Lu et al., 1992; Krouk et al., 2011).

Regulation of carbon and nitrogen is tightly linked to plant-environment interactions and it determine plant growth and development (Raven et al., 2004). Thus, to gain more insights into the modulation of shoot growth, BNF, N and carbon metabolism and tissue composition elicited by externally applied N in *S. spontaneum* accessions G152 and G3, we analysed the expression of genes involved in carbon, N, amino acid and hormone metabolism in both accessions grown under externally supplied N (**Figure 9**). Here, we first discuss some of the general but important trends in gene expression as affected by N application, followed by more specific findings related to the inter-linked processes of carbon, N, amino acids and hormone metabolism. Overall, N application significantly altered >2000 genes in both accessions combined, with genotype accounting for most of the variation (**Figure S1, S2**). This suggests that large genetic variation in gene expression exists between G152 and G3, and that G152 is relatively more sensitive to applied N. This contention is further corroborated by the very small number of common DEGs (28) found between the two accessions when their N treatment effects were compared (**Figure S2**). However, in contrast to treatment effect (LN *vs* HN for each accession) where G152 had almost thrice the number of DEGs detected in G3, the number of DEGs were similar when both accessions grown under same N condition (HN or LN) were compared (**Figure S1, S2**). Also, there was remarkable similarity of classes of DEGs associated with different cellular activities under LN and HN as shown in **Figures 8C, D**. However, it is interesting to note that more genes were found to be up-regulated under low N whereas high N had an opposite effect (**Figure S1**). Contrasting expression of genes involved in plant nutrition under different nutrient availability has been reported previously. For instance, under low N growth condition high- affinity N transporters are strongly up-regulated whereas they are down-regulated when N is replete (Dreyer and Michard, 2020). An opposite expression pattern occurs for low-affinity N transporters.

External application of inorganic N markedly up- and down-regulated genes in all metabolic pathways studied (**Figures 7**–**9**). More specifically, the set of genes involved in N, carbon, amino acid and hormone metabolism showed remarkably varied expression patten in different genotypes and N supply condition (**Figure 9**), giving further evidence of the complexity and variation in molecular mechanism regulating these pathways in different genetic background. A clearer picture of differential gene expression has emerged with GO and KEGG analyses. GO analysis of DEGs revealed very large difference in gene expression pattern between G152 and G3 (**Figure 7**). In G152, applied N caused remarkable effect on carbon and N metabolism and transport processes with more up-regulation than down-regulation, while single organism processes and lipid and carboxylic acid metabolism were the most affected (more down- than up-regulation) in G3. It appears that under low N, activity of genes associated with phosphorylation, kinase activity and metabolism of phosphate-containing compounds are more affected (more down- than up-regulation). In contrast, under HN, expression of genes involved in nucleotide binding, kinase activity, DNA metabolism are more altered (more up- than down-regulation) (**Figure 7**). These results were further corroborated by the KEGG analysis of DEGs, with amino acid, carbohydrate and N metabolism dominating in G152 in response to N and DEGs of lipid metabolism enriching far in excess than others in G3 (**Figure 8**).

The overall picture arising from the molecular analysis is that the two accessions may be achieving the same outcome of relatively high shoot growth (**Table 1**) and remarkable resilience of BNF (**Figure 3**) possibly through different metabolic strategies (**Figures 7**, **8**). Uptake, transport and use of N and other minerals needed for plant growth involve considerable energy. It appears that G152 may be directly utilising photosynthate to drive N assimilation and use (**Figure 8**) whereas G3 may be relying more on lipids for the same (**Figure 8**). Further, the molecular evidence points towards a remarkable difference in expression of a large number of same genes or their alleles associated with carbon, N, amino acid and hormone metabolism in different genetic background and contrasting N supply conditions.

## 5 Conclusion

Reducing N fertiliser use for sugarcane production is critical for reducing soil acidification and carbon footprint. While optimisation of cropping system will help improve the environmental sustainability of sugarcane production, replicating the Brazilian experience of low N input farming using varieties with BNF capacity that complement improved cropping system would be a very desirable outcome. Until now, efforts to breed or select N-efficient sugarcane varieties proved unsuccessful, suggesting the genetic complexity of sugarcane NUE and possibly the limited BNF capacity in the current breeding pool. In this context, exploring sugarcane progenitor *S. spontaneum*, the species with very large eco-climatic adaptability including very low-fertile marginal soils, to breed for N-efficient clones would be a logical approach. Our *S. spontaneum* screening experiments provided the first evidence of large genetic variation for BNF capacity existing in this species. Further, BNF property of a small number of clones proved to be quite resilient to external inorganic N application, without which economic crop production remains unattainable. Molecular characterization of high-BNF accessions unraveled the diversity of gene activity and metabolic pathways associated with carbon, N, amino acid and hormone metabolism operating in different *S. spontaneum* accessions. Understanding the genetic elements and the molecular mechanism(s) underpinning BNF in *S. spontaneum* would be the next logical step for research. It is concluded that *S. spontaneum* accessions with high BNF capacity could be a valuable tool to improve N-efficiency in sugarcane.

## Data availability statement

The datasets presented in this study can be found in online repositories. The names of the repository/repositories and accession number(s) can be found in the article/**Supplementary Material**.

## Author contributions

TL conceived, designed, and conducted research, analysed data and wrote the original manuscript. PL and C-NL contributed methodology. PL, TL, RY and X-YL analysed data and wrote the final manuscript. KH conducted experimental work. Y-RL contributed to original manuscript writing. X-YL revised the manuscript. PL and Y-RL reviewed and edited original manuscript. All authors contributed to the article and approved the submitted version.
