# Supplementary material for: Physiological and molecular insights into the resilience of biological nitrogen fixation to applied nitrogen in Saccharum spontaneum, wild progenitor of sugarcane
Source: Front Plant Sci. 2023 Jan 13;13:1099701. doi: 10.3389/fpls.2022.1099701 (PMC9881415; doi:10.3389/fpls.2022.1099701)
Supplement: Supplementary file 1 [file DataSheet_1.zip › CONTRIBUTION OF THIS WORK TO THE FIELD OF RESEARCH.DOCX]

**Physiological and molecular insights into the resilience of biological nitrogen fixation in *Saccharum spontaneum*, wild progenitor of sugarcane**

Ting Luo^1*^, Chang-ning Li^1^, Rui Yan^1^, Kejun Huang^1^, Yang-rui Li^1^, Xiaoyan Liu^1^, Prakash Lakshmanan^1, 2, 3*^

**CONTRIBUTION OF THIS WORK TO THE FIELD OF RESEARCH**

The work presented in the manuscript is scientifically novel and significant from the perspectives of environment and sustainable production of sugarcane, the leading bioenergy and sugar crop globally. The research reported here falls within the scope of the Frontiers in Plant Science (FIPS) special issue topic “Nitrogen Use Efficiency: Plant Biology to Crop Improvement”. The scientific novelty and practical importance of the work here is detailed below.

Sugarcane accounts for >80% of sugar and about 35% of bioethanol produced in the world, and it is grown in >110 countries. It being a rapidly growing high biomass crop, high fertiliser (N, P and K) input is commonplace in many countries, causing serious environmental issues. Considering the growing pressure on reducing carbon footprint from agriculture, substantial effort is now underway in developing nitrogen use efficient sugarcane, but with limited success so far. In this context we are exploring the opportunity to exploit biological nitrogen fixation (BNF) inherent in sugarcane wild progenitor species to improve N use in this crop. In the current study we report three scientifically novel and important findings

1. The first report of large genotypic variation for BNF in sugarcane progenitor species *Saccharum spontaneum* germplasm collection
2. Identification of *Saccharum spontaneum* accessions with high BNF capacity, which is highly resilient to external nitrogen fertiliser application- this is an important finding scientifically and from variety development perspective
3. Remarkable genetic variation for carbon, nitrogen, amino acid and hormone metabolism-related gene activity in high BNF clones, indicating that different molecular and metabolic strategies related to carbon and nitrogen metabolism may be operating in different *Saccharum spontaneum* accessions

This work clearly expands our understanding of BNF and N use in the progenitor of an important food and energy crop (sugarcane) and report germplasm suitable for introgression breeding with high BNF that is resilient to external inorganic N.

We confirm that neither the manuscript nor any parts of its content are currently under consideration or published in another journal.
