# Supplementary material for: Physiological and molecular insights into the resilience of biological nitrogen fixation to applied nitrogen in Saccharum spontaneum, wild progenitor of sugarcane
Source: Front Plant Sci. 2023 Jan 13;13:1099701. doi: 10.3389/fpls.2022.1099701 (PMC9881415; doi:10.3389/fpls.2022.1099701)
Supplement: Supplementary file 1 [file DataSheet_1.zip › Supplementary Figures.DOCX]

**
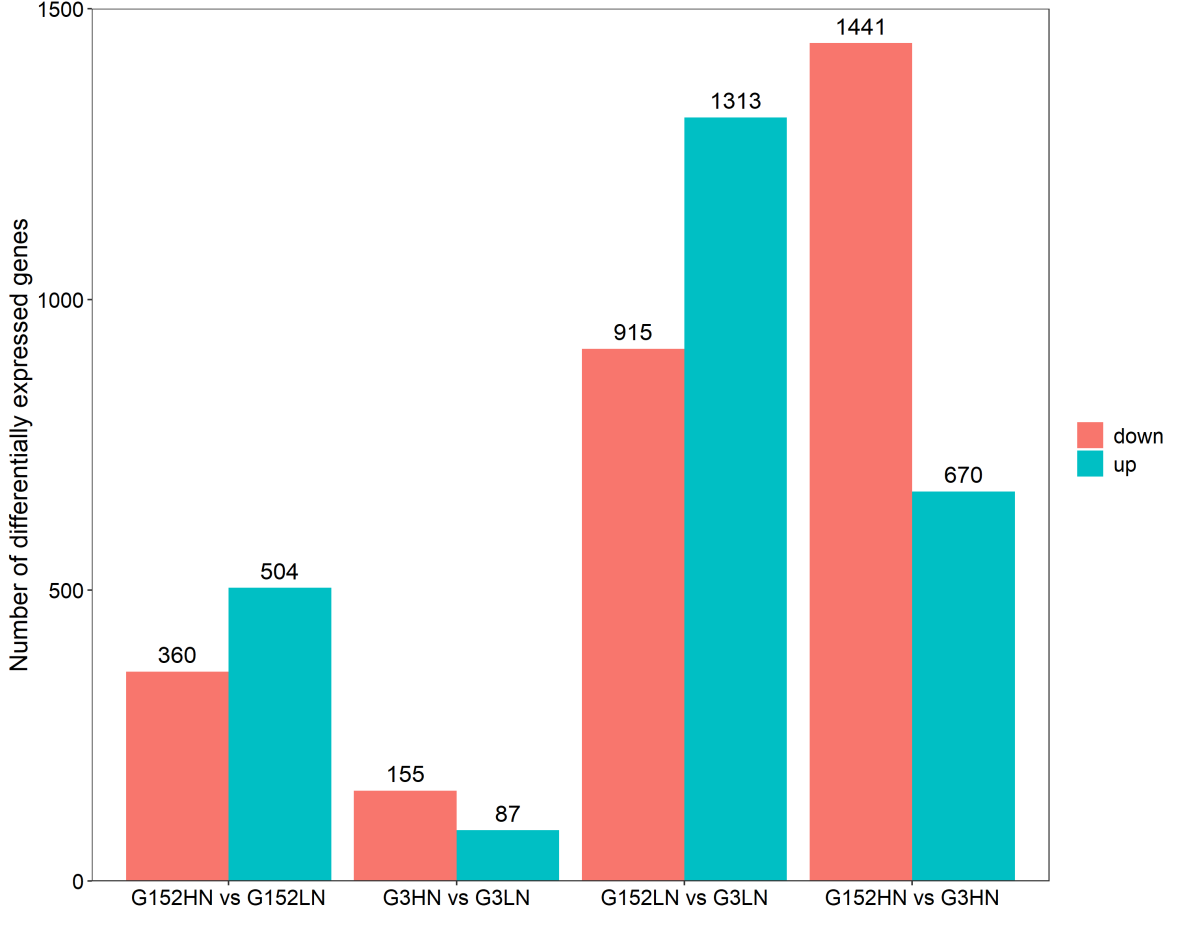
Figure S1 Effect of external N supply and genotype on differential gene expression in leaves of S. spontaneum accessions G152 and G3**


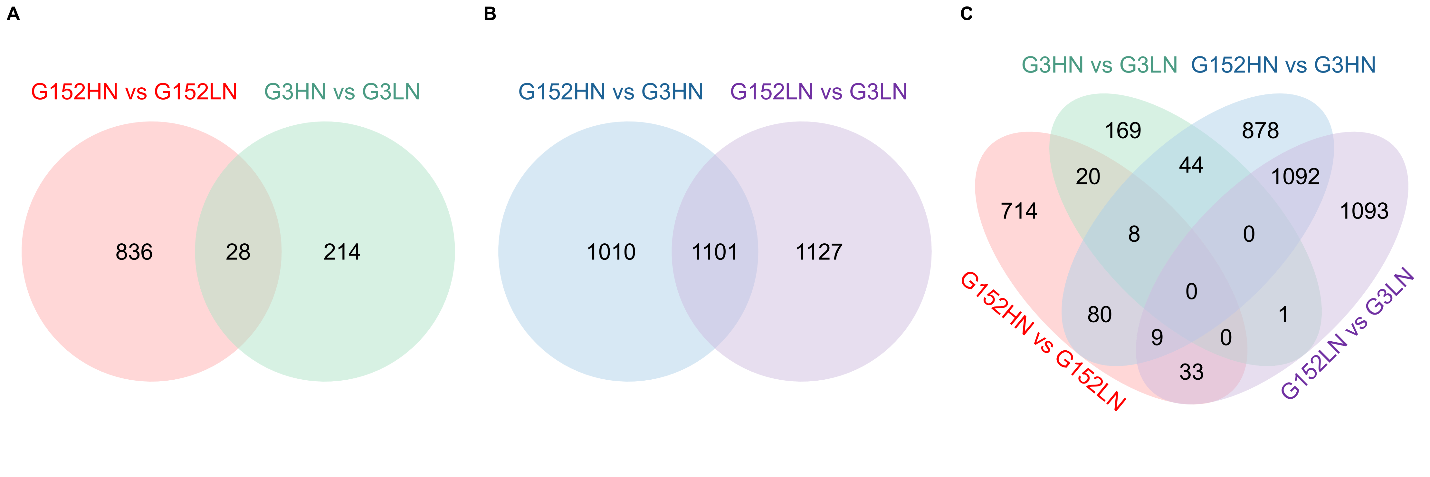


**Figure S2. Venn diagrams of common and unique DEGs identified from pair-wise comparisons of S. spontaneum accessions G152 and G3 treated with N. HN= high N, LN= low N.**
