# Supplementary material for: Physiological and molecular insights into the resilience of biological nitrogen fixation to applied nitrogen in Saccharum spontaneum, wild progenitor of sugarcane
Source: Front Plant Sci. 2023 Jan 13;13:1099701. doi: 10.3389/fpls.2022.1099701 (PMC9881415; doi:10.3389/fpls.2022.1099701)
Supplement: Supplementary file 1 [file DataSheet_1.zip › Supplementary Table 1.DOCX]

**Physiological and molecular insights into the resilience of biological nitrogen fixation in *Saccharum spontaneum*, wild progenitor of sugarcane**

Ting Luo^1*^, Chang-ning Li^1^, Rui Yan^1^, Kejun Huang^1^, Yang-rui Li^1^, Prakash Lakshmanan^1, 2, 3*^

^1^Sugarcane Research Institute, Guangxi Academy of Agricultural Sciences; Key Laboratory of Sugarcane Biotechnology and Genetic Improvement (Guangxi), Ministry of Agriculture and Rural Affairs; Guangxi Key Laboratory of Sugarcane Genetic Improvement, Guangxi Academy of Agricultural Sciences, Nanning 530007, China.

^2^Interdisciplinary Research Center for Agriculture Green Development in Yangtze River Basin, College of Resources and Environment, Southwest University, Chongqing 400716, China

^3^Queensland Alliance for Agriculture and Food Innovation, University of Queensland, St Lucia 4067, QLD, Australia.

**Correspondence**:

Ting Luo: [seafair@163.com](mailto:seafair@163.com); ORCID ID 0000-0003-0427-479X

Prakash Lakshmanan: [plakshmanan2018@outlook.com](mailto:plakshmanan2018@outlook.com); ORCID ID [0000-0003-1725-0391](https://orcid.org/0000-0003-1725-0391?lang=en)

**Table S1.**

**1. NCBI submission details**

Accession number: PRJNA847754

Submission ID: SUB11589278

Release date: 2022-06-10

SRA records will be accessible with the following link after the indicated release date:

<https://www.ncbi.nlm.nih.gov/sra/PRJNA847754>

**Summary of RNASeq data**

| Unigenes | Number of transcripts | Number of genes |
| --- | --- | --- |
| 200-500 bp | 110271 | 27404 |
| 500- 1000 bp | 62468 | 41099 |
| 1k-2k bp | 58526 | 28295 |
| >2kb | 34963 | 14149 |
| Total | 266228 | 110947 |
| Minimum length (bp) | 201 | 201 |
| Mean length (bp) | 996 | 1087 |
| Maximum length (bp) | 17867 | 17867 |
| N50 | 1613 | 1466 |
| N90 | 418 | 533 |
| GC% | 53.5 |  |
