# Supplementary material for: Physiological and molecular insights into the resilience of biological nitrogen fixation to applied nitrogen in Saccharum spontaneum, wild progenitor of sugarcane
Source: Front Plant Sci. 2023 Jan 13;13:1099701. doi: 10.3389/fpls.2022.1099701 (PMC9881415; doi:10.3389/fpls.2022.1099701)
Supplement: Supplementary file 1 [file DataSheet_1.zip › Supplementary Table 9.DOCX]

| Gene ID | Forward primer | Reverse primer | Product size (bp) | Annotation |
| --- | --- | --- | --- | --- |
| c43527_g3_i1 | CATCACTGGAACGACACGAATC | GGTCTCTTAACTACACGCTGGA | 147 | Amino acid metabolism |
| c45156_g2_i0 | CGTCGTCACCGCTTACTCT | GCAGCCTACTCCGAGAAGATT | 175 |  |
| c46870_g3_i0 | CCTCCTCTTCTCTTCTCGTCTC | GGATTGGACTGGACTGTTGGT | 192 |  |
| c47126_g2_i | GTGGACACGGTAGTTGTAGGAG | CGCAGCAGCACCAGTTCTA | 140 | Carbon metabolism |
| c66987_g0_i0 | ATCCTTCACTTGGCGAGTTCAT | GCACATCAGCAACCATACTTCC | 198 |  |
| c47582_g0_i1 | GATTGCCGACGACTACCACTC | GCCTGTCCACTCATTGCTCTT | 118 |  |
| c59722_g4_i0 | ACAGCCAGATTGTTCCAGCAT | GCCACAGCCTCAGTTCCTAC | 175 |  |
| c51974_g0_i0 | TCACAAGCACACGACAACAAG | TAATGAGCCGAGACGAAGTCAA | 107 | Nitrogen metabolism |
| c56112_g2_i0 | CCATCAACGCCATTACCACAC | CAGAAGCACCAGCACCAGTA | 181 |  |
| c55068_g0_i0 | CGTGGACAGTGTGCTTGGA | CGGCGGAGGACTACTACAAC | 138 |  |
| GAPDH | AGGACTCCAAGACCCTCCTC | CTTCTTGGCACCACCCTTCA | 170 | Reference genes |
| ACAD | CGTGGCATGGATCTGATGGT | AGCCTGCTCCAGTTCAATCC | 120 |  |
| CAC | ACAACGTCAGGCAAAGCAAA | AGATCAACTCCACCTCTGCG | 112 |  |

**Supplementary Table S9. Primers of genes used for qRT-PCR validation**
